# miRNA-183-5p.1 promotes the migration and invasion of gastric cancer AGS cells by targeting TPM1

**DOI:** 10.3892/or.2020.7902

**Published:** 2020-12-16

**Authors:** Jun Lin, Juan Shen, Hao Yue, Zhongwei Cao

Oncol Rep 42: 2371-2381, 2019; DOI: 10.3892/or.2019.7354

Following the publication of the above article, an interested reader drew to the authors’ attention that an apparent duplication of data panels had occurred in Fig. 4; essentially, the pre-NC/GES-1 and pre-NC/MKN-7 panels in Fig. 4A and C, respectively, appeared very similar to each other, with the exception of different values reported for the gated percentages. The authors consulted their original data and were able to determine that the error arose inadvertently during the process of compiling the figure.

The revised version of Fig. 4, featuring the corrected data for the pre-NC/MKN-7 panel in Fig. 4C, is shown on the next page. The authors have confirmed that the errors associated with this figure did not have any significant impact on either the results or the conclusions reported in this study, and are grateful to the Editor of *Oncology Reports* for allowing them the opportunity to publish this Corrigendum. Furthermore, they apologize to the readership of the Journal for any inconvenience caused.

## Figures and Tables

**Figure 4.**
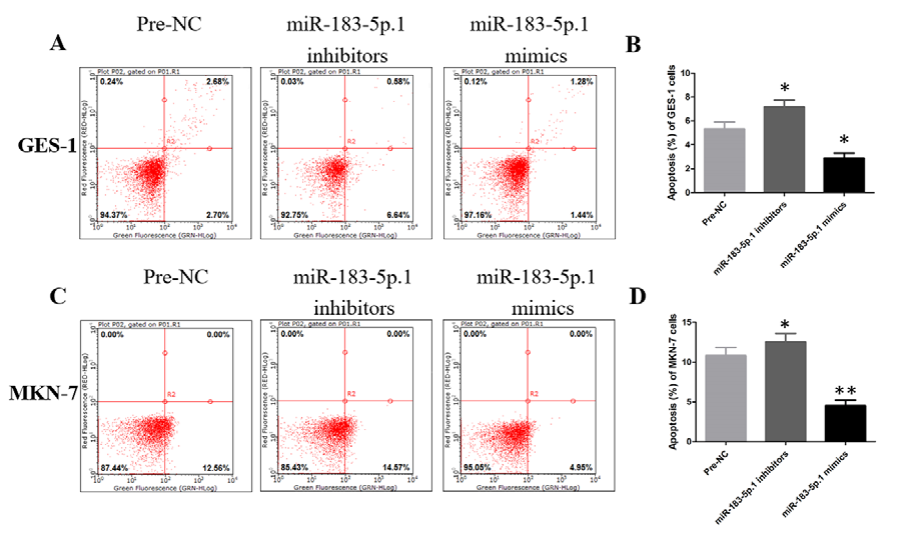

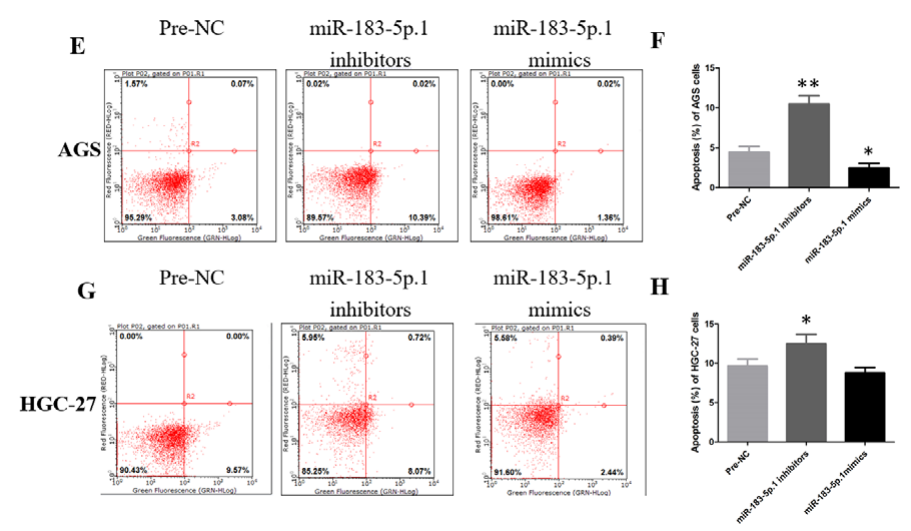
miR-183-5p.1 regulates the apoptosis of GC cells as detected by flow cytometry. miR-183-5p.1 inhibitors increased the apoptosis of (A and B) GES-1, (C and D) MKN-7, (E and F) AGS and (G and H) HGC-27 cells, while miR-183-5p.1 mimics significantly inhibited the apoptosis of (A and B) GES-1, (C and D) MKN-7, (E and F) AGS and (G and H) HGC-27 cells. Results are presented as the mean ± standard error of the mean (n=3). *P<0.05, **P<0.01. GC, gastric cancer; miR, microRNA.

